# Improved Phenotype-Based Definition for Identifying Carbapenemase Producers among Carbapenem-Resistant *Enterobacteriaceae*

**DOI:** 10.3201/eid2109.150198

**Published:** 2015-09

**Authors:** Nora Chea, Sandra N. Bulens, Thiphasone Kongphet-Tran, Ruth Lynfield, Kristin M. Shaw, Paula Snippes Vagnone, Marion A. Kainer, Daniel B. Muleta, Lucy Wilson, Elisabeth Vaeth, Ghinwa Dumyati, Cathleen Concannon, Erin C. Phipps, Karissa Culbreath, Sarah J. Janelle, Wendy M. Bamberg, Alice Y. Guh, Brandi Limbago, Alexander J. Kallen

**Affiliations:** Centers for Disease Control and Prevention, Atlanta, Georgia, USA (N. Chea, S.N. Bulens, T. Kongphet-Tran, A.Y. Guh, B. Limbago, A.J. Kallen);; Minnesota Department of Health, St. Paul, Minnesota, USA (R. Lynfield, K.M. Shaw, P. Snippes Vagnone);; Tennessee Department of Health, Nashville, Tennessee, USA (M.A Kainer, D.B. Muleta);; Maryland Emerging Infections Program, Baltimore, Maryland, USA (L. Wilson, E. Vaeth);; New York–Rochester Emerging Infections Program, Rochester, New York, USA (G. Dumyati, C. Concannon);; University of Rochester Medical Center, Rochester (G. Dumyati, C. Concannon);; University of New Mexico, Albuquerque, New Mexico, USA (E.C. Phipps, K. Culbreath);; Colorado Department of Public Health and Environment, Denver, Colorado, USA (S.J. Janelle, W.M. Bamberg)

**Keywords:** carbapenemase, carbapenem-resistant, carbapenemase producers, Enterobacteriaceae, CRE, enterobacteria, enterobacterial infection, bacteria, antimicrobial resistance, identification, surveillance, prevention, United States, Emerging Infections Program, EIP

## Abstract

Preventing transmission of carbapenemase-producing, carbapenem-resistant *Enterobacteriaceae* (CP-CRE) is a public health priority. A phenotype-based definition that reliably identifies CP-CRE while minimizing misclassification of non–CP-CRE could help prevention efforts. To assess possible definitions, we evaluated enterobacterial isolates that had been tested and deemed nonsusceptible to >1 carbapenem at US Emerging Infections Program sites. We determined the number of non-CP isolates that met (false positives) and CP isolates that did not meet (false negatives) the Centers for Disease Control and Prevention CRE definition in use during our study: 30% (94/312) of CRE had carbapenemase genes, and 21% (14/67) of *Klebsiella pneumoniae* carbapenemase–producing *Klebsiella* isolates had been misclassified as non-CP. A new definition requiring resistance to 1 carbapenem rarely missed CP strains, but 55% of results were false positive; adding the modified Hodge test to the definition decreased false positives to 12%. This definition should be considered for use in carbapenemase-producing CRE surveillance and prevention.

## Abstract 2

A new, less restrictive definition increases detection of *Klebsiella pneumoniae* carbapenemase producers.

Multidrug-resistant organisms are a major public health concern worldwide (*1*–*4*). Of particular concern has been the emergence of resistance to carbapenem antimicrobial drugs among *Enterobacteriaceae* (*4*,*5*). In the United States, the reported percentage of common health care–associated infections caused by carbapenem-nonsusceptible *Enterobacteriaceae* increased from 1.2% in 2001 to 4.2% in 2011 (*4*), and the greatest increase (≈10%) occurred among *Klebsiella* species (*4*).

Although carbapenem nonsusceptibility among *Enterobacteriaceae* can result from several mechanisms, much of the recent increase in carbapenem-resistant *Enterobacteriaceae* (CRE) in the United States is likely due to the spread of carbapenemase-producing strains, particularly *Klebsiella* species that produce *Klebsiella pneumoniae* carbapenemase (KPC) (*3*,*4*). In addition to KPC, several other carbapenemases have been identified in the United States: New Delhi metallo-β-lactamase (NDM), oxacillinase (OXA), Verona integron–encoded metallo-β-lactamase (VIM), and imipenemase (IMP) (*5*,*6*). These enzymes are encoded by mobile genetic elements that have the potential to spread between bacterial species. The uptake of these elements among different bacterial species could result in further increases in the prevalence of carbapenem-resistant or panresistant bacteria, or both, and if this occurs, treatment options in the United States would be limited (*7*). Since 2006, the Centers for Disease Control and Prevention (CDC) has identified >100 NDM-producing CRE in the United States, including those that caused 2 hospital-based outbreaks (*8*,*9*). In light of the elements described above, much of the effort to prevent further spread of CRE has targeted carbapenemase-producing CRE. However, these efforts have been hampered because many clinical laboratories do not routinely perform CRE resistance-mechanism testing, so they cannot differentiate carbapenemase-producing CRE from CRE that are carbapenem-nonsusceptible due to other mechanisms. In addition, resistance-mechanism testing is also not routinely recommended for clinical purposes by the Clinical and Laboratory Standards Institute (CLSI) (*10*).

A phenotype-based CRE definition (i.e., based on antimicrobial drug susceptibility pattern) that is specific for carbapenemase-producing strains has the potential to facilitate CRE prevention by allowing health care facilities to target these strains for the most aggressive interventions without the need to rely on resistance-mechanism testing. The pre-2015 CDC CRE surveillance definition—nonsusceptiblity to imipenem, meropenem, or doripenem, and resistance to all third-generation cephalosporins tested, as determined by using CLSI M100-S23 testing standards (*11*)—was originally designed to preferentially identify carbapenemase-producing CRE (*9*). However, because of the number of antimicrobial drugs included and the complexity of the third-generation cephalosporin restriction (resistance to all tested), this phenotype-based definition proved to be complicated and difficult to implement by health care facilities for both surveillance and infection control efforts. In addition, use of this definition led to the mistaken assumption that CRE that did not meet the definition did not warrant any additional infection control precautions beyond standard precautions (*9*).

The objective of this analysis was to identify a phenotype-based definition that accurately differentiates carbapenemase-producing CRE from non–carbapenemase-producing CRE on the basis of antimicrobial susceptibility patterns. To achieve this, we evaluated isolates collected through CDC’s Emerging Infections Program (EIP) CRE surveillance system (http://www.cdc.gov/hai/eip/mugsi.html).

## Methods

### Inclusion Criteria and Data Collection

Isolates of *Enterobacter* spp., *Escherichia coli, and Klebsiella* spp. were collected from clinical laboratories that serve 6 EIP sites in the United States: Minnesota and Tennessee (both statewide); the 5-county Denver, Colorado, metropolitan area (Arapahoe, Adams, Denver, Douglas, and Jefferson Counties); the 4-county Baltimore, Maryland, metropolitan area (Baltimore City, Baltimore County, Howard County, and Carroll County); the Albuquerque, New Mexico, metropolitan area (Bernalillo County); and the Rochester, New York, metropolitan area (Monroe County). Four sites (Colorado, Maryland, New Mexico, and New York) submitted isolates from a preselected group of laboratories during March 10, 2013–January 30, 2014; two sites (Minnesota and Tennessee) submitted isolates received from statewide reporting starting January 1, 2011, and continuing through January 30, 2014. If >1 isolate of the same genus was obtained from a single patient, only 1 was included. Isolates that met the following 3 criteria were included: 1) evidence of nonsusceptibility (intermediate or resistant) to any carbapenem (imipenem, meropenem, doripenem, or ertapenem), as determined on the basis of susceptibility testing conducted at the local clinical laboratory by using 2013 CLSI breakpoints (*11*); 2) availability of susceptibility testing data from the reporting clinical laboratory for all antimicrobial drugs tested in the assessed phenotype-based definitions (Table 1); and 3) documentation of methods used for susceptibility testing.

**Table 1:** Summary of 11 phenotype-based definitions evaluated for reliability in identifying carbapenemase producers among carbapenem-resistant *Enterobacteriaceae*, United States, January 1, 2011–January 30, 2014*

Antimicrobial included	Study inclusion criteria	Definition†										
		1	2	3	4	5	6	7	8	9	10	11
Any carbapenem‡	NS				R		R	R		NS§	R	
Any carbapenem (without ertapenem)		NS	NS	NS		R						NS
>2 carbapenems‡									NS§			
All third-generation cephalosporins tested			R				R					
Any third-generation cephalosporins tested				R				R				
Cefepime										R	R	R

*NS, nonsusceptible; R, resistant. Blank cells mean not included in the definition. †Interpretation based on Clinical and Laboratory Standards Institute breakpoints (M100-S23) (*11*). Definitions: 1, nonsusceptible to any carbapenem, excluding ertapenem; 2, nonsusceptible to any carbapenem, excluding ertapenem, and resistant to all third-generation cephalosporins tested (pre-2015 Centers for Disease Control and Prevention carbapenem-resistant Enterobacteriaceae surveillance definition); 3, nonsusceptible to any carbapenem, excluding ertapenem, and resistant to any third-generation cephalosporins tested; 4, resistant to any carbapenem; 5, resistant to any carbapenem, excluding ertapenem; 6, resistant to any carbapenem and resistant to all third-generation cephalosporins tested; 7, resistant to any carbapenem and resistant to any third-generation cephalosporin tested; 8, nonsusceptible to at least 2 carbapenems (ertapenem resistant, if tested); 9, nonsusceptible to any carbapenem (ertapenem resistant, if tested) and resistant to cefepime; 10, resistant to any carbapenem and resistant to cefepime; and 11, nonsusceptible to any carbapenem, excluding ertapenem, and resistant to cefepime. ‡Ertapenem, doripenem, imipenem, and meropenem. §If ertapenem used in the definition, isolate would need to be resistant (i.e., MIC >2 μg/mL).

### Confirmatory Testing at CDC

Eligible *Enterobacter* spp., *E. coli, and Klebsiella* spp. isolates were sent to CDC for reference susceptibility testing (broth microdilution and Kirby-Bauer disk diffusion testing) for ertapenem, doripenem, imipenem, meropenem, 3 third-generation cephalosporins (ceftriaxone, cefotaxime, and ceftazidime), and cefepime (*11*). Three methods were used to evaluate each isolate for the presence of carbapenemases: the modified Hodge test (MHT), a broth microdilution screening test for metallo-β-lactamases that compares the MIC of imipenem in the presence and absence of metal chelators (*12*), and PCR for the most common carbapenemases in the United States (i.e., *bla*_KPC_, *bla*_NDM,_ and *bla*_OXA-48_). Isolates that were *bla*_NDM_-negative by PCR but *bla*_NDM_-positive by metallo-β-lactamase screening were further evaluated by PCR for *bla*_VIM_ and *bla*_IMP_.

### Analysis

Eleven phenotype-based definitions (Table 1) were initially evaluated: 1) nonsusceptible to any carbapenem, excluding ertapenem; 2) nonsusceptible to imipenem, meropenem, or doripenem and resistant to all third-generation cephalosporins tested (pre-2015 CDC CRE surveillance definition); 3) nonsusceptible to any carbapenem, excluding ertapenem, and resistant to any third-generation cephalosporins tested; 4) resistant to any carbapenem; 5) resistant to any carbapenem, excluding ertapenem; 6) resistant to any carbapenem and resistant to all third-generation cephalosporins tested; 7) resistant to any carbapenem and resistant to any third-generation cephalosporin tested; 8) nonsusceptible to at least 2 carbapenems (ertapenem resistant, if tested); 9) nonsusceptible to any carbapenem (ertapenem resistant, if tested) and resistant to cefepime; 10) resistant to any carbapenem and resistant to cefepime; and 11) nonsusceptible to any carbapenem, excluding ertapenem, and resistant to cefepime. All susceptibility interpretations were determined on the basis of the 2013 CLSI breakpoints (*11*). With the exception of CRE that are OXA-48–like producers, most carbapenemase producers are multidrug resistant and should be resistant to third-generation cephalosporins. Thus, in an attempt to improve detection of carbapenemase-producing CRE, we included third-generation cephalosporins in certain definitions. Similarly, we added cefepime to certain definitions to ascertain if it might help discriminate between AmpC-producing and carbapenemase-producing CRE.

For each of the 11 phenotype-based definitions, we performed 4 calculations based on the clinical laboratory–determined susceptibility results for carbapenem-nonsusceptible isolates. The calculations determined the number and percentage of 1) carbapenemase-producing isolates that screened positive (true positives [TP]); 2) carbapenemase-producing isolates identified that screened negative (selected false negatives [sFNs]); 3) non–carbapenemase-producing isolates that screened positive (false positives [FPs]); and 4) non–carbapenemase-producing isolates identified that screened negative (selected true negative [sTN]). The denominator for each of the calculations was the number of isolates for which the definitions could be applied on the basis of results at the clinical laboratory. Because we limited our isolates to those with nonsusceptibility to a carbapenem and could only calculate sFN and sTN screening results, we could not determine the specificity, sensitivity, or negative predictive value of a definition. Three of the 11 definitions were further stratified by EIP site and organism tested to evaluate differences in their FP and sFN results by geographic region and by genus. The 3 definitions were the one that obtained the lowest number of sFNs, the one that obtained the lowest number of FPs among definitions with potentially acceptable levels of sFNs (defined as <10%), and the pre-2015 CDC CRE surveillance definition. Analysis was limited to EIP sites that submitted >50 isolates. We performed 2-step testing by adding MHT results to the susceptibility results for the isolates meeting the definition with the lowest number of sFNs to determine if the results of the MHT affected the the percentage of isolates classified as FP and sFN.

## Results

A total of 312 isolates were included in this evaluation; the number from each EIP site and the number for each included genus are shown in Table 2. A carbapenemase gene was identified in 94 (30%) of the 312 isolates. Seventy-two (65%) *Klebsiella* spp. isolates had a carbapenemase gene, of which 67 (93%) were KPC and 5 (7%) were NDM. Of all *Enterobacter* spp. and *E. coli* isolates, 14 (14%) and 8 (8%), respectively, had a carbapenemase gene, and all were KPC. The percentage of carbapenemase-producing CRE at the various sites was 73% in Maryland (40 [93%] KPC, 3 [7%] NDM); 30% in Minnesota (31 [94%] KPC, 2 [6%] NDM); 20% in Tennessee (13 [100%] KPC); 6% in New York (3 [100%] KPC); 7% in New Mexico (1 [100%] KPC); and 0 in Colorado.

**Table 2:** Isolates used in a study evaluating phenotype-based definitions for reliability in identifying carbapenemase producers among carbapenem-resistant enterobacterial isolates from 6 US Emerging Infections Program sites, January 1, 2011–January 30, 2014

Site	No. (%) isolates			Total no. isolates, N = 312
	*Klebsiella* spp., n = 111	*Enterobacter* spp., n = 103	*Escherichia coli*, n = 98	
Minnesota	30 (27)	63 (56)	19 (17)	112
Tennessee	17 (25)	11 (16)	41 (59)	69
Maryland	48 (81)	0	11 (19)	59
New York	11 (20)	20 (38)	22 (42)	53
New Mexico	5 (33)	6 (40)	4 (27)	15
Colorado	0	3 (75)	1 (25)	4

The numbers and percentages of FPs and sFNs obtained with each of the 11 evaluated definitions are shown in Table 3. The percentage of FPs and sFNs ranged from 5.5% to 55.0% and 0.7% to 27.7%, respectively. The 3 phenotype-based definitions meeting the requirements for the prespecified stratified analysis by site and genus were the one with the lowest number of sFNs (definition 4, resistant to any carbapenem); the one with the lowest number of FPs among definitions with potentially acceptable levels of sFNs, defined as <10% (definition 5, resistant to any carbapenem without ertapenem); and the pre-2015 CDC CRE surveillance definition (definition 2).

**Table 3:** False-positive and selected false-negative results in a study evaluating phenotype-based definitions for reliability in identifying carbapenemase producers among carbapenem-resistant enterobacterial isolates from 6 US Emerging Infections Program sites, January 1, 2011–January 30, 2014

Result	No. isolates/no. tested (%), by definition no., N = 307*										
	1	2	3	4	5	6	7	8	9	10	11
False-positive	117/307 (38.1)	82/307 (26.7)	91/307 (29.6)	169/307 (55.0)	57/307 (18.6)	146/307 (47.6)	153/307 (49.8)	60/307 (19.5)	37/307 (12.1)	34/307 (11.1)	17/307 (5.5)
Selected false-negative	12/307 (3.9)	15/307 (4.9)	13/307 (4.2)	2/307 (0.7)	17/307 (5.5)	7/307 (2.3)	4/307 (1.3)	27/307 (8.8)	85/307 (27.7)	85/307 (27.7)	85/307 (27.7)

*False-positive isolates are those meeting the definition but not found to produce a carbapenemase. Selected false-negative isolates were selected on the basis of nonsuceptibility to >1 carbapenem not meeting the definition but found to produce a carbapenemase. Definitions: 1, nonsusceptible to any carbapenem, excluding ertapenem; 2, nonsusceptible to any carbapenem, excluding ertapenem, and resistant to all third-generation cephalosporins tested (pre-2015 Centers for Disease Control and Prevention carbapenem-resistant Enterobacteriaceae surveillance definition); 3, nonsusceptible to any carbapenem, excluding ertapenem, and resistant to any third-generation cephalosporins tested; 4, resistant to any carbapenem; 5, resistant to any carbapenem, excluding ertapenem; 6, resistant to any carbapenem and resistant to all third-generation cephalosporins tested; 7, resistant to any carbapenem and resistant to any third-generation cephalosporin tested; 8, nonsusceptible to at least 2 carbapenems (ertapenem resistant, if tested); 9, nonsusceptible to any carbapenem (ertapenem resistant, if tested) and resistant to cefepime; 10, resistant to any carbapenem and resistant to cefepime; and 11, nonsusceptible to any carbapenem, excluding ertapenem, and resistant to cefepime.

The numbers and percentages of FPs and sFNs obtained by using these 3 definitions are shown by EIP site in Table 4. The percentage of FPs was highest in Minnesota and Tennessee, and the percentage of sFNs was highest in Tennessee. The number and percentage of FPs and sFNs obtained by using the same 3 definitions are shown by organism tested in Table 5. The highest percentage of sFNs obtained by using definitions 2 and 5 were among *Klebsiella* spp.; overall, sFNs were generally lower for *E. coli* and *Enterobacter* spp. Of note, definition 4 had the narrowest variability in the percentage of sFNs across all sites (range 0%–1.5%) and among the 3 enterobacterial organisms (range 0%–1.1%). Of the 67 KPC-producing *Klebsiella* spp., 14 (21%), 1 (1%), and 14 (21%) did not meet definitions 2, 4, and 5, respectively. Of the 14 KPC-producing *Klebsiella* spp. isolates that did not meet definitions 2 and 5, a total of 12 (86%) were susceptible to all carbapenems tested except ertapenem. All 5 NDM-producing *Klebsiella* spp. met the 3 definitions.

**Table 4:** Results, by study site, for select phenotype-based definitions used to identify carbapenemase producers among 307 carbapenem-resistant enterobacterial isolates from 4 US EIP, Emerging Infections Program sites, January 1, 2011–January 30, 2014*

Site	No. isolates/no. tested (%), by definition no.†										
	2‡			4§			5¶			4 plus MHT#	
	FP	sFN		FP	sFN		FP	sFN		FP	sFN
Minnesota	51/111 (45.9)	3/111 (2.7)		55/111 (49.5)	1/111 (0.9)		25/111 (22.5)	5/111 (4.5)		23/111 (20.7)	1/111 (0.9)
Tennessee	17/65 (26.2)	4/65 (6.2)		50/65 (76.9)	1/65 (1.5)		18/65 (27.7)	4/65 (6.2)		3/65 (4.6)	1/65 (1.5)
Maryland	6/59 (10.2)	5/59 (8.5)		16/59 (27.1)	0/59		3/59 (5.1)	6/59 (10.2)		3/59 (5.1)	0/59
New York	4/53 (7.5)	2/53 (3.8)		31/53 (58.5)	0/53		8/53 (15.1)	1/53 (1.9)		3/53 (5.7)	0/53

*FP, false positive; MHT, the modified Hodge test; sFN, selected false negative. †False-positive isolates are those meeting the definition but not found to produce a carbapenemase. Selected false-negative isolates were selected on the basis of nonsuceptibility to >1 carbapenem not meeting the definition but found to produce a carbapenemase. ‡Definition 2 nonsusceptible to any carbapenem, excluding ertapenem, and resistant to all third-generation cephalosporins tested (pre-2015 Centers for Disease Control and Prevention carbapenem-resistant Enterobacteriaceae surveillance definition). §Definition 4, resistant to any carbapenem. This definition obtained the lowest number of selected false-negatives. ¶Definition 5, resistant to any carbapenem, excluding ertapenem. This definition obtained the lowest number of false-positives among definitions with selected false-negatives of <10%. #Definition 4 (resistant to any carbapenem) plus MHT (i.e., 2-step testing).

**Table 5:** Results, by organism tested, for select phenotype-based definitions used to identify carbapenemase producers among 307 carbapenem-resistant enterobacterial isolates from 6 US Emerging Infections Program sites, January 1, 2011–January 30, 2014*

Organism	Definition no., result, no. isolates/no. total (%)†										
	2‡			4§			5¶			4 plus MHT#	
	False-positive	Selected false-negative		False-positive	Selected false-negative		False-positive	Selected false-negative		False-positive	Selected false-negative
*Klebsiella* spp.	15/111 (13.5)	14/111 (12.6)		31/111 (27.9)	1/111 (0.9)		11/111 (9.9)	14/111 (12.6)		3/111 (2.7)	1/111 (0.9)
*Enterobacter* spp.	42/102 (41.2)	0/102		68/102 (66.7)	0/102		26/102 (25.5)	0/102		30/102 (29.4)	0/102
*Escherichia coli*	25/94 (26.6)	1/94 (1.1)		70/94 (74.5)	1/94 (1.1)		20/94 (21.3)	3/94 (3.2)		3/94 (3.2)	1/94 (1.1)

*MHT, the modified Hodge test. †False-positive isolates are those meeting the definition but not found to produce a carbapenemase. Selected false-negative isolates were selected on the basis of nonsuceptibility to >1 carbapenem not meeting the definition but found to produce a carbapenemase. ‡Definition 2 nonsusceptible to any carbapenem, excluding ertapenem, and resistant to all third-generation cephalosporins tested (pre-2015 Centers for Disease Control and Prevention carbapenem-resistant Enterobacteriaceae surveillance definition). §Definition 4, resistant to any carbapenem. This definition obtained the lowest number of selected false-negatives. ¶Definition 5, resistant to any carbapenem, excluding ertapenem. This definition obtained the lowest number of false-positives among definitions with selected false-negatives of <10%. #Definition 4 (resistant to any carbapenem) plus MHT (i.e., 2-step testing).

A comparison of the MHT and PCR results by enterobacterial organism and carbapenem used in the MHT is shown in Table 6. The MHT showed no sFNs for all 3 organisms and a small number of FPs for *Klebsiella* spp. (3%) and *E. coli* (3%–4%); however, the MHT misclassified 31%–34% of non–carbapenemase-producing *Enterobacter* spp. as carbapenemase producers. The effect from adding the MHT to definition 4 is shown in Tables 4 and 5. Addition of the MHT to definition 4 decreased the overall percentage of FPs from 55% to 12%, but the percentage of sFNs remained at 0.7%. FPs were reduced substantially for *Klebsiella* spp. (from 27.9% to 2.7%) and *E. coli* (74.5% to 4%) but remained higher for *Enterobacter* spp. (29%).

**Table 6:** Results for modified Hodge test evaluation of 312 enterobacterial isolates from 6 US Emerging Infections Program sites, January 1, 2011–January 30, 2014

Organism, carbapenem used	False-positive results, %	Selected false-negative results
*Klebsiella* spp.		
Meropenem	2.7	0
Ertapenem	2.7	0
*Enterobacter* spp.		
Meropenem	31	0
Ertapenem	34	0
*Escherichia coli*		
Meropenem	3	0
Ertapenem	4	0

## Discussion

In this evaluation, no phenotype-based definition identified all carbapenemase-producing CRE without also capturing a substantial number of non–carbapenemase-producing CRE. The percentages of FPs and sFNs varied by enterobacterial organism and by EIP site, likely due to the underlying variation in the prevalence of carbapenemase-producing CRE in different areas and among different *Enterobacteriaceae*. In this sample of isolates, the pre-2015 CDC CRE surveillance definition misclassified nearly 13% of carbapenem-nonsusceptible *Klebsiella* spp. isolates and 21% of KPC-producing *Klebsiella* spp. isolates as non–carbapenemase producing. In light of this finding, a phenotype-based definition that captures all (or nearly all) carbapenemase-producing CRE should be considered for surveillance and prevention. However, our data demonstrate that alternative definitions that accomplish this also increase the number of FPs and thus have the potential to increase the amount of work and the cost associated with CRE surveillance and prevention efforts.

Current efforts to control CRE in the United States have used infection prevention strategies targeted at carbapenemase-producing strains; however, most clinical laboratories do not routinely differentiate carbapenemase-producing from non–carbapenemase-producing strains. Molecular detection of genes encoding carbapenemases is the reference standard for identifying carbapenemase-producing CRE, but this testing requires substantial expertise and expense. More readily available tests, like the MHT, could likely be performed in most clinical microbiology laboratories, but they require additional technician time and reagents, which creates a burden on laboratory resources and therefore limits their routine use. In addition, the MHT might falsely identify NDM-producing strains as non–carbapenemase-producing CRE and might falsely identify non–carbapenemase-producing *Enterobacter* spp. as carbapenemase-producing CRE (*13*). Another carbapenemase detection test, the Carba-NP, has good performance characteristics and may be a viable alternative; however, it is not yet widely used (*13*–*16*). Because of the limited availability and technical challenges associated with resistance-mechanism testing for CRE, a definition for CRE that increases detection of carbapenemase-producing strains while reasonably limiting the number of non–carbapenemase-producing strains identified would aid surveillance and infection control efforts until resistance-mechanism–based testing becomes more routinely available.

Our results show that the use of definition 4 (resistant to any carbapenem) obtained one of the lowest percentages of sFN results. In addition, between EIP sites and between the 3 enterobacterial organisms, there was little variability in the percentage of isolates with sFN results, suggesting the results may be reflective of what other hospitals in the United States might experience when using this CRE definition to capture carbapenemase-producing CRE isolates. In January 2015, CDC modified its surveillance definition for CRE. The change was made partly because of the results of findings from this evaluation but also as an effort to simplify the CRE surveillance definition so that it can be applied more easily. The new definition (resistant to imipenem, meropenem, doripenem, or ertapenem or documentation that the isolate possesses a carbapenemase) is to be used with current CLSI breakpoints (*10*). To further reduce the number of non–carbapenemase-producing CRE strains falsely identified as carbapenemase-producing CRE, health care facilities could consider adding resistance-mechanism testing for isolates that meet this definition. Such testing may be particularly helpful in areas with a low prevalence of CRE and with organisms that are less likely to produce carbapenemases (e.g., *E. coli* and *Enterobacter* spp.).

This evaluation has several limitations. First, our testing collection consisted of a relatively small number of isolates from a limited number of sites, and because strain typing was not performed on any of the isolates included in this analysis, we cannot exclude the possibility that some of these isolates might have been related to each other. However, this evaluation did include isolates from diverse locations in the United States that represent areas with low and relatively high prevalences of CRE. Second, isolates from only 3 genera were included, limiting the generalizability of any conclusions beyond these organisms. Last, our sample included mostly KPC-producing CRE among the carbapenemase-producing strains. These results may not be applicable to other emerging carbapenemases, specifically NDM and OXA. However, current epidemiology suggests that KPC remains the most common carbapenemase in the United States.

In conclusion, the pre-2015 CDC CRE surveillance definition failed to identify some carbapenemase-producing strains. A definition that includes only resistance to any 1 of the 4 approved carbapenems is simpler and misses fewer carbapenemase-producing strains, but at the cost of increasing FPs. The addition of the MHT to this definition further limits FPs; however, this testing is not routinely used in the United States. In general, all organisms that are nonsusceptible to a carbapenem are potentially multidrug-resistant and, at minimum, warrant the use of interventions such as contact precautions to minimize transmission. Health care facilities could choose to reserve more aggressive interventions, such as screening of contacts and patient cohorting, for patients with isolates that meet this new definition, which appears to more completely detect carbapenemase-producing CRE. Health care facilities wishing to limit the work and expense associated with more aggressive interventions could perform resistance-mechanism testing on isolates meeting this new definition and apply interventions only when the isolates are confirmed to produce carbapenemase.
